# The activation of the atypical PKC zeta in light-induced retinal degeneration and its involvement in L-DNase II control

**DOI:** 10.1111/jcmm.12539

**Published:** 2015-03-17

**Authors:** Imene Jaadane, Sabine Chahory, Chloé Leprêtre, Boubaker Omri, Laurent Jonet, Francine Behar-Cohen, Patricia Crisanti, Alicia Torriglia

**Affiliations:** a INSERM U1138, Centre de Recherches des Cordeliers, Université Paris Descartes, Université Pierre et Marie CurieParis, France; b ENVA, Ecole Nationale Vétérinaire d’AlfortMaison Alfort, Paris, France; c Hôpital Ophtalmique Jules-GoninLausanne, Switzerland

**Keywords:** PKC zeta, serpin B1, retinal apoptosis, retinal degeneration, light

## Abstract

Light-induced retinal degeneration is characterized by photoreceptor cell death. Many studies showed that photoreceptor demise is caspase-independent. In our laboratory we showed that leucocyte elastase inhibitor/LEI-derived DNase II (LEI/L-DNase II), a caspase-independent apoptotic pathway, is responsible for photoreceptor death. In this work, we investigated the activation of a pro-survival kinase, the protein kinase C (PKC) zeta. We show that light exposure induced PKC zeta activation. PKC zeta interacts with LEI/L-DNase II and controls its DNase activity by impairing its nuclear translocation. These results highlight the role of PKC zeta in retinal physiology and show that this kinase can control caspase-independent pathways.

## Introduction

Genetic and oxidative stresses may damage the retina leading to vision loss and, in dramatic cases, to blindness. Environmental factors such cigarette smoke, exposure to harmful substances and even to intense light, are important in the progression of many retinopathies 1. There are good evidence supporting the fact that the light is a factor producing retinal damages depending on radiation intensity and on time of exposure 2,3. Many models have been developed to study and evaluate the light toxicity (or phototoxicity, defined as the thermal, chemical, or mechanical damage to the retina resulting of an exposure to visible or ultraviolet light) on the retina and to elucidate the mechanisms of oxidative stress-induced death of photoreceptors. These models allowed a better understanding and identification of the molecular process involved in degenerative retinal damages observed in various retinopathies, because they present a synchronized death of photoreceptors. Light-induced retinal degeneration (LIRD) has been largely used to identify apoptosis as a mechanism of photoreceptors cell death 4. In models of retinal degeneration, where weak intensity of light and long-term exposure were used, the classical effectors of apoptosis are not activated 5–8, instead, a caspase-independent pathway, the leucocyte elastase inhibitor (LEI)/L-DNase II mediates photoreceptor demise 9,10.

Leucocyte elastase inhibitor is an ubiquitous protease inhibitor of the serpin family (serine protease inhibitor) 11. It is a cytoplasmic protein bearing an antiprotease activity. After cleavage by its cognate protease LEI undergoes a conformational change that allows the exposure of both an endonuclease active site and a nuclear localization signal (NLS) 12,13. In this way LEI is transformed into L-DNase II (LEI-derived DNase II) which is translocated to the nucleus, where it cleaves DNA generating oligonucleosome fragments bearing 3′P ends 14. We have previously shown that LEI/L-DNase II has opposite activities regarding apoptosis: in its native state, LEI has an anti-apoptotic function by preventing of proteolysis 15,16, whereas in its cleaved form it is pro-apoptotic by degrading genomic DNA 15,17.

Atypical protein kinase C (PKC) zeta is a serine threonin kinase involved in cell protection against various stresses 18. Our laboratory has shown that this kinase is activated in the retina following intravitreal administration of tumour necrosis factor (TNF) 19 and that is cytoprotective in endotoxin-induced uveitis (EIU) 20. As many PKCs, it is a cytoplasmic protein which is translocated to the plasma membrane or to the nucleus upon activation 21,22. This activation can be modulated by its pseudo-substrate domain 23 or by endogenous inhibitors such as prostate apoptosis response 4 (PAR4) that hides the catalytic domain of the kinase 24. It has been shown that following stress, activated PKC zeta phosphorylates Ikappa kinase B, which leads, to the release of Nuclear Factor Kappa B (NFkB) and its nuclear translocation. NFkB promotes the transcription of anti-apoptotic genes such as Bcl-2, inhibitor of apoptosis protein, TNF receptor-associated factors 25. So that, activation of PKC zeta and NFkB pathways act as inhibitory mechanisms of cell death in stress response.

In this study, we show that PKC zeta is activated in LIRD. We investigated if this activation is inhibiting the L-DNase II pathway formerly involved in this retinal degeneration.

## Materials and methods

### Retinal light damage in rats

Six to eight-week-old Fischer male rats were exposed to a constant light and sacrificed as described before 9.

### Intravitreal injection of PKC zeta inhibitor

The rats were anaesthetized by an intramuscular injection of xylazin (Rompon®; Bayer Pharma, Puteaux, France) at 10 mg/kg and ketamine (Ketamine Virbac 500®; Vibrac France, Carros, France) at 40 mg/kg. A local anaesthesia is performed by an instillation of oxybuprocaine (Cebesine®; Laboratoire Chauvin (Bausch and Lomb) Montpellier, France). The intravitreal injection of PKC zeta inhibitor is carried out under a surgical microscope using a 30 G needle mounted on an insulin syringe (BD Micro-Fine, Becton Dickinson S.A., Le Pont de Claix, France). PKC zeta inhibitor is injected at a concentration of 6 μg per eye in a volume of 10 μl.

### Protein extraction from illuminated retina

After defrosting, the retinas were homogenized in 20% weight-volume of the M-PER extraction reagent (Thermo Scientific 78503, Illkirch, France), using a pellet pestle motor homogenizer (KONTES) on ice. The homogenate was then incubated on ice for 15 min. and then centrifuged (15,000 × g, 4°C) for 15 min. Supernatant was recovered for Western blot analysis.

### Preparation of cryosections

Enucleated eyes were embedded in optimal cutting-temperature medium (Tissue Tek compound Sakura) cooled with liquid nitrogen. Cryosections of 10 μm were prepared by using a cryostat (Leica CM 3050S, Nanterre, France) and stored at −20°C until immunohistochemical analysis.

### Cell lines and culture conditions

Baby hamster kidney (BHK) and HeLa cells (S3 clone) were cultured as described before 26.

### Cell death induction

HeLa cells were seeded at a density of 20,000 cells/cm^2^, maintained in culture for 2 days and then treated for 7 hrs with TNFα at 50 ng/ml (Gibco, Life Technologies, Saint Aubin, France) and 10 μg/ml of cycloheximide (CHX) (Sigma-Aldrich, Saint-Quentin Fallavier, France). Baby hamster kidney cells were treated with 5-(*N*,*N*-hexamethylene) amiloride (HMA; stock solution: 40 mM in DMSO, A9561; Sigma-Aldrich) at 40 μM for 18 hrs.

### Pull-down assay

Recombinant proteins (wild-type LEI and calmodulin) were produced as before 26. Samples were dosed and resolved by SDS-PAGE and immunoblotted for PKC zeta (C20, sc-216; Santa Cruz Biotechnology, Clinisciences, Nanterre, France). Calmodulin was used as a negative control and a crude extract of HeLa cells was used as a positive control.

### Co-immunoprecipitation

Co-immunoprecipitation was performed with the ProFound™ Mammalian Co-Immunoprecipitation Kit (Pierce, Thermo Fischer Scientific, Courtaboeuf, France) as before 26. The eluted material was then denatured in Laemmli buffer and analysed by Western blot with LEI or PKC zeta antibody. Column charged without IgG was used as a negative control.

### Immunocytochemistry

HeLa cells were seeded in coverslips at the density of 5 × 10^4^ cells/ml in 2 ml of complete medium using 12-multiwell plates. Forty-eight hours after seeding they were treated with 50 ng/ml of TNFα plus 10 μg/ml of CHX for 7 hrs. After treatment they were washed twice with PBS containing Ca^2+^ and Mg^2+^, fixed with 4% paraformaldehyde during 15 min. (all steps were performed at room temperature) and then washed twice with PBS. Permeabilization was performed with 0.3% Triton X100 in PBS for 20 min. Then, the cells were washed twice with PBS. Non-specific protein binding sites were blocked by 1 hr incubation in a blocking buffer containing 1% non-fat milk in PBS. Cells were then incubated with anti-P-NFkB (Abcam ab51059, Paris, France), anti-PKC zeta or anti-LEI/L-DNase II 1/100, in 0.1% non-fat milk in PBS for 1 hr. This was followed by two washes with PBS and incubation for 1 hr with a 1/500 dilution of anti-rabbit Alexa Fluor® 546 conjugated antibody, or anti-rabbit Alexa Fluor® 488 conjugated antibody, or anti-chicken Alexa Fluor® 488 conjugated antibody (Invitrogen, Life Technologies, Saint Aubin, France). Cells were then washed twice with PBS, incubated for 1 min. with 4-6 di-aminidino-2-phenyl indoledichloride (DAPI) 1/5000 (Sigma-Aldrich) and washed three times with PBS. Finally, coverslips were mounted with fluoromount (Sigma-Aldrich, Saint-Quentin Fallavier, France). Immunoreactivity was visualized using fluorescence microscopy with an Olympus microscope BX51 (Olympus France, Rungis, France).

### Western blot

After extraction of samples (retina or cells) with M-PER, protein measurement was performed with bicinchoninic acid (BCA)™ Protein Assay Kit (Thermo Scientific) using bovine serum albumin as standard. After dilution of samples with Laemmli buffer, 25 μg of the extracted proteins was separated by a 12% SDS-PAGE, immobilized on nitrocellulose membrane (PROTAN®, Whatman®, GE Healthcare, Versailles, France) and blotted with: anti-PKC zeta, anti-phospho-PKC zeta (Santa Cruz sc-12894R; Santa Cruz Biotechnology), anti-PAR4 (Santa Cruz sc-1807; Santa Cruz Biotechnology), anti-actin (Santa Cruz sc-1616; Santa Cruz Biotechnology), anti-lamin B (Santa Cruz sc-6216; Santa Cruz Biotechnology) at 1/500 dilution or anti-LEI/L-DNase II at 1/1000 dilution. The secondary antibodies conjugated to HRP (Vector, Eurobio, Les Ulis, France) were used in a 1/5 000 dilution. Finally Luminata Forte Western HRP Substrate (Millipore, Merck Chimie, Fontenay sous Bois, France) was used to reveal the signal.

### Site-directed mutagenesis

Site-directed mutagenesis was performed on wild-type (wt) LEI conjugated to pDsRed 12 using the Quick-Change mutagenesis kit from Stratagene according to the manufacturer’s protocol. A threonine residue located in position 195 was changed into a glutamate, and the mutant was called T195E. Baby hamster kidney cells were seeded at a density of 5 000 cells/cm^2^ on glass coverslips in 24-well plates. Two hours before transfection, the culture medium was replaced with fresh medium without foetal calf serum. The transfection medium contained 0.4 μg of pDsRed-T195E-LEI mutant, 0.4 μg of wt LEI coupled to GFP 12 and 1 μl of Lipofectin reagent (Life Technologies, Inc., Saint Aubin, France) per well. The transfection process occurred at 37°C for 5 hrs and was stopped by adding the same volume of DMEM containing 20% foetal calf serum. Coverslips were collected 24 hrs after transfection and put in fresh medium containing or not 40 μM of HMA for 18 hrs. Images were taken using a fluorescence microscopy.

### Knockdown of PKC zeta by siRNA transfection

To perform knockdown experiments, HeLa cells were transfected with 50 nM of siRNA PKC zeta Smart pool (Thermo Scientific L-003526-00-0005), or control siRNA (Santa Cruz sc-36869; Santa Cruz Biotechnology) with AMAXA® cell line nucleofector kit. Transfected cells were seeded in four wells from a 24-well plate. After 17 hrs, cells were treated with TNFα and CHX, as before, for 7 hrs. Finally, a MTT (Thiazolyl Blue Tetrazolium Bromide) reduction assay or a preparation of subcellular fractions was performed.

### Viability assay based on MTT reduction

Thiazolyl Blue Tetrazolium Bromide (MTT) is a colorimetric assay based on the ability of oxidoreductase enzymes to reduce the tetrazolium. In our conditions, the MTT assay reflected cell viability. After 7 hrs of treatment with TNFα/CHX, culture medium was removed and 250 μl of MTT solution was added to each well (MTT from Sigma-Aldrich was diluted in PBS at a concentration of 1 mg/ml). The plate was kept for 1 hr at 37°C. Cells were then lysed with 250 μl of isopropanol. MTT reduction in each sample was subsequently assessed by measuring absorption at 570 nm *versus* 630 nm using a microplate reader (Bio-Rad, Marnes-la-Coquette, France).

### Preparation of subcellular fractions

For detection of LEI/L-DNase II redistribution after treatment, both the cytosolic and the nuclear fractions were isolated from cells. 10^6^ HeLa cells were transfected (or not) with siRNA PKC zeta by AMAXA® cell line nucleofector then seeded in six well plates for 17 hrs. After treatment with TNFα/CHX for 7 hrs, attached cells were collected by cell scrapping using a rubber policeman, floating cells were collected by centrifugation of the culture medium. Cells were pooled and washed two times in cold PBS (every time at 370 × g, 5 min. at 4°C). Cells were then collected and resuspended in an ice-cold hypo-osmotic solution of 1.5 mM MgCl_2_. After mechanical breakage of cell membranes by using a tight-fit Dounce B potter (15 strokes) on ice, they were centrifuged and the cytoplasmic fractions contained in the supernatants were collected. The pellets were washed two times with the same MgCl_2_ solution and resuspended in 100 μl of m-PER extract reagent (Pierce). This was considered as the nuclear fraction. Finally, the protein concentration in the cytoplasmic and nuclear fractions, was performed with the BCA method™ Protein Assay Kit (Thermo Scientific). Bovine serum albumin was used as standard. Laemmli buffer was added for further Western blot analysis.

### Endonuclease activity assay

Retinal extracts (15 μg) were incubated at 37°C with 1.5 μg of pHook plasmid in 20 mM Tris-EDTA buffer pH 5.5. 10 μl of the reaction was sampled at different times. The samples were analysed on a 1% agarose gel containing ethidium bromide (BET).

## Results

### Activation of PKC zeta in LIRD

**Figure 1:**
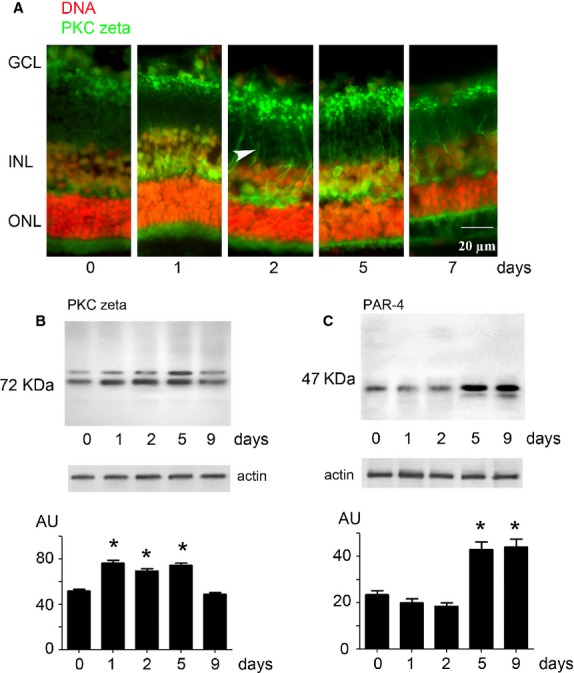
PKC zeta in retina during LIRD. (A) Rats were exposed to a continuous white light for 1–7 days, and then killed; eyes were mounted in optimal cutting-temperature (OCT). Cryosections were stained with anti-PKC zeta (green) and with propidium iodide (DNA, red). Arrow head shows translocation of PKC zeta to the plasma membrane. ONL: outer nuclear layer; INL: inner nuclear layer; GCL: ganglion cell layer. Scale bar represents 20 μm. (B) Western blot analysis of PKC zeta expression at different times of light exposure. Specificity of the antibody was verified by competition with the specific peptide (sc-216P). Lower panel shows the quantification; means statistically different with respect to control (*P* < 0.01 (anova test)) are indicated by an ‘*’. (C) same experiment as in B using anti-PAR-4 antibody. Specificity of the antibody was verified by competition with the specific peptide (sc-4216). ‘*’ indicates significant differences with respect to the control, as before. Actin was used as a charge control.

To investigate the cellular mechanisms involved in retinal protection during light exposure, we studied the status of PKC zeta. As other PKCs, this kinase is mobilized to the cell membrane when activated. So that, we analysed retinas from rats exposed to white light from different periods, by immunohistochemistry, using an anti-PKC zeta as staining antibody (Fig.1A). On day 1, we observed a staining of PKC zeta in the outer and inner nuclear layers. On day 2, an intense labelling appeared at extensions of cells localized in the inner portion of the retina (from the outer plexiform layer to the ganglion cell layer). Although this staining of the extensions was still seen at 7 days of exposure, the PKC zeta expression seemed to decrease after 7 days of light exposure.

To verify if the change in PKC zeta localization after illumination was because of an increase in protein expression, we analysed extracts from whole retinas by Western blot (Fig.1B). We observed that native PKC zeta (72 kDa) increased from 1 to 5 days of light exposure compared to retinas from rats kept in normal light conditions. After 9 days, expression of PKC zeta decreased to the control level. Different from other experimental settings 21,27, only a major band of 72 kDa was observed in all conditions. No cleavage of PKC zeta was found.

Protein kinase C zeta is, in general, transiently activated. This activation is stopped by the release of the stress or by the induction of cell death. If a caspase-dependent apoptosis pathway is activated, PKC zeta is cleaved. As no fragment was found, we investigated if its endogenous inhibitor, PAR-4 was synthesized. Figure1C shows the level of PAR-4 expression in neural retina after illumination. A weak decrease in PAR-4 expression was seen at 1 and 2 days followed by an important increase at 5 and 9 days. The late increase in PAR-4 expression together with the decrease in PKC zeta expression suggested that this pathway was shutting down.

### The interaction between LEI/L-DNase II and PKC zeta

**Figure 2:**
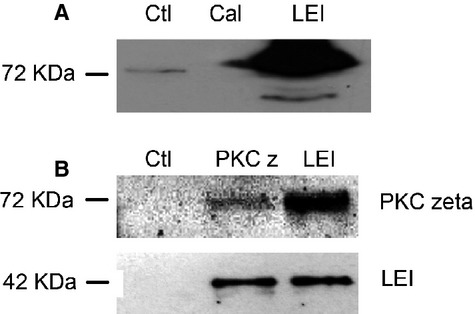
Physical interaction between LEI/L-DNase II and PKC zeta. (A) Pull down of PKC zeta using a LEI affinity column. An His-cartridge resin was loaded with an His-tagged LEI. Crude extract of 100 μg from HeLa cells was loaded on the column that was then washed and eluted. The LEI line represents the material eluted from this column. As a negative control an his-tagged calmodulin was used as naïve protein. Ctl line represents 10 μg of HeLa crude extract. (B) Crude extract of 100 μg from HeLa cells was immunoprecipitated with anti-PKC zeta (PKC z line) or anti-LEI (LEI line). A column without IgG was used as control (Ctl). Western blots were developed with anti-PKC zeta (upper panel) or anti-LEI (lower panel). These are representative experiments out of three.

We have already shown that the caspase-independent pathway, LEI-L-DNase II, is involved in photoreceptor cell death 9 during LIRD. To investigate if PKC zeta was a possible modulator of this pathway we investigated if this kinase could interact with LEI/L-DNase II. As we presumed that this interaction, if it exists, could be a general phenomenon not limited to the retina, we used cells in culture for these experiments, to save as many rodents as possible. Routinely we used both HeLa and BHK cells, which are cells from different species (human and hamster respectively) and from different origin (epithelial cancer cells and fibroblasts respectively), to avoid cell-specific interactions. We first conducted pull-down experiments using a purified recombinant protein bearing an His-tag in its N-terminal end and loaded on His-select cartridges. Figure2A shows a Western blot of a HeLa cell extract after elution from a LEI-loaded column, developed with an anti-PKC zeta antibody. ‘Ctl’ represents the PKC zeta signal obtained from 10 μg of total cell extract. ‘LEI’ indicates the material obtained from elution of an LEI affinity column. A nickel resin charged with an His-tagged calmodulin was used as the negative control. Western blot revealed an intense PKC zeta band indicating that the LEI/L-DNase II protein was able to bind PKC zeta *in vitro*.

To further confirm this interaction co-immunoprecipitations of the proteins of interest were performed on BHK and HeLa cells extracts. Here again, different types of cells were used to avoid cell-specific interactions. Western blot analysis revealed that PKC zeta was co-immunoprecipitated with an anti-LEI antibody and that LEI was co-immunoprecipitated with an anti-PKC zeta antibody. Any protein was detected by antibodies in the column loaded without IgG (negative control) (Fig.2B).

Taken together these results indicated that LEI and PKC zeta could associate to each other.

### The control of the LEI/L-DNase II pathway by phosphorylation

**Figure 3:**
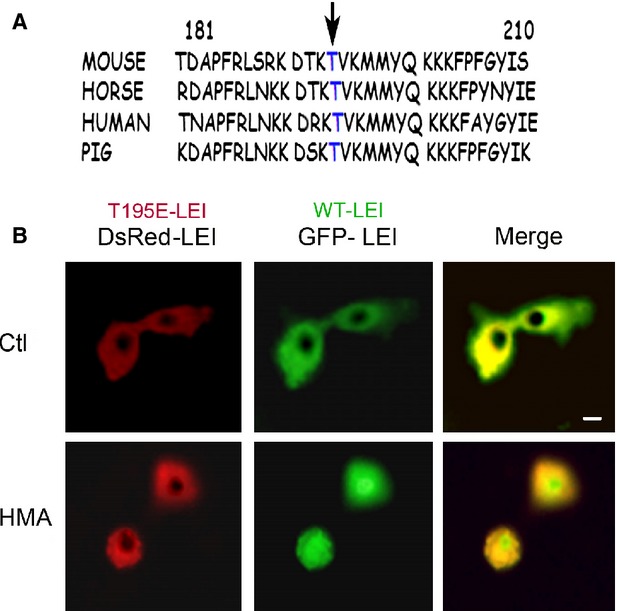
Putative phosphorylation site of LEI. (A) Fragment of the sequence of LEI presenting the consensus site for PKC zeta. LEI from several species are aligned showing that the sequence is well conserved among species. (B) GFP-LEI (WT) was transfected to BHK cells together with pDsRed-T195E-LEI (mutant protein). Cells were left untreated (Ctl panel) or treated with HMA to induce apoptosis. Nuclear translocation of the mutant protein is impaired. Scale bar represents 10 μm.

To investigate if the LEI/L-DNase II pathway could be regulated by PKC zeta, we looked for a putative PKC target sequence on LEI/L-DNase II. *In silico* studies revealed the presence of a putative phosphorylation site near by the NLS of LEI. In fact, the threonine 195 located in the ‘linker’ sequence between the two lysine’s groups of the NLS (Fig.3A) corresponded to a consensus sequence of the PKC phosphorylation substrate 28. Using site-directed mutagenesis, we substituted this threonine by a glutamate to mimic a constitutive phosphorylation (the negative charge simulated the addition of a phosphate group). The T195E mutation was made on a DsRed-LEI chimera protein (red fluorescent protein). A GFP-LEI chimera (green protein) was left unchanged and used as an internal control (wt LEI).

Next, BHK cells were cotransfected with these two proteins. 48 hrs after transfection, the cells were left untreated or induced in apoptosis with 40 μM of HMA for 18 hrs. Subsequently, cells were analysed by fluorescence microscopy (Fig.3B). In control conditions, the two proteins had the same cytoplasmic localization. However, after induction of cell death by HMA, the T195E mutant, mimicking a phosphorylation, was not translocated into the nucleus in contrast to the wild type (WT) protein. These results suggested that the pseudo threonine phosphorylation could control the nuclear translocation of L-DNase II during apoptosis.

### The effect of LEI-PKC zeta interaction on cell survival *in vitro*

To estimate the functional impact of LEI/L-DNase II-PKC zeta interaction on cell survival, we raised the hypothesis that under stress conditions, PKC zeta was activated, resulting in the L-DNase II phosphorylation. This phosphorylation could impair or slow down the nuclear translocation of L-DNase II. In this way, the PKC zeta activation could exert its protective role.

As this issue was very difficult to study in the retina, we looked for a cellular model of cell death where the activation of PKC zeta and LEI/L-DNase II pathways were already described. We selected as a model the treatment of HeLa cells with TNFα (50 ng/ml) and CHX (10 μg/ml) for 7 hrs, a model described by Smith and Smith 29.

**Figure 4:**
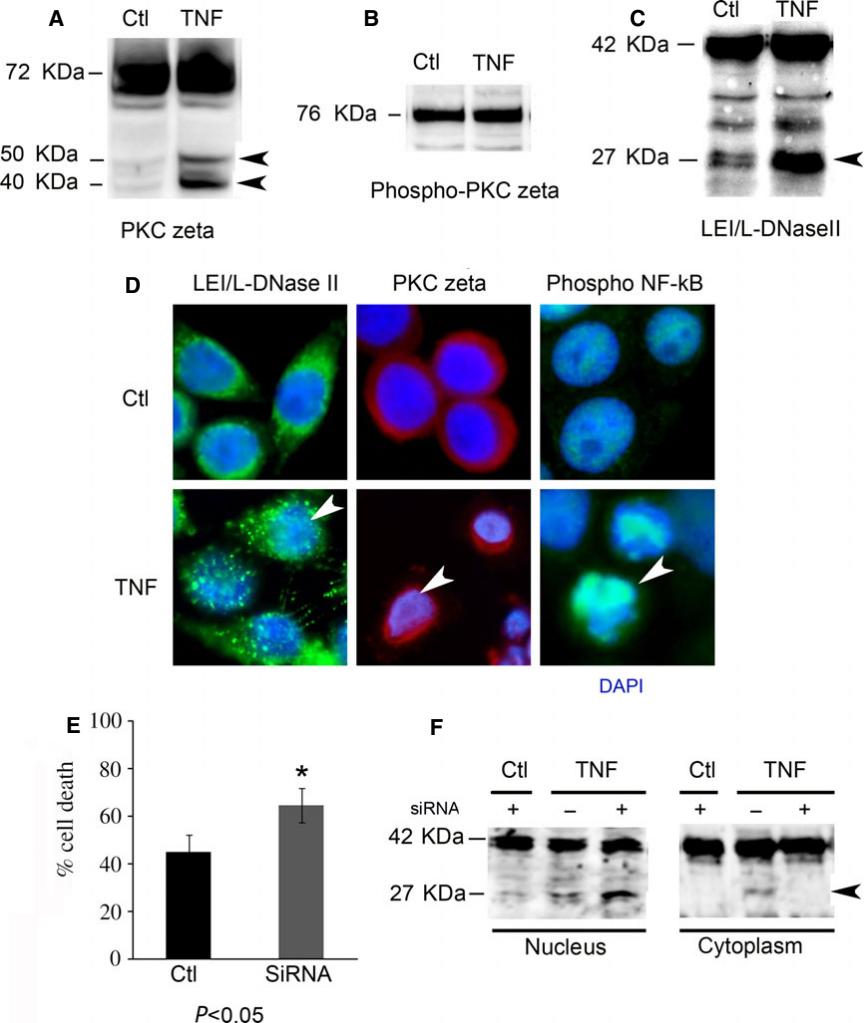
L-DNase II control by PKC zeta in HeLa cells. HeLa cells were induced in apoptosis by TNFα/cycloheximide. (A) Western blot analysis of PKC zeta in total extract of HeLa cells treated with TNFα/CHX (TNF) or left untreated (Ctl). Arrow heads indicate the fragments of PKC zeta. (B) Western blot analysis of serine 311 phosphorylated PKC zeta in TNFα/CHX treated or untreated cells (Ctl) The specificity of the antibody was performed by digesting the protein extract with 1 IU of calf intestinal alkaline phosphatase, invitrogen 18009-019. (C) Western blot analysis of LEI/L-DNase II in total extract of HeLa cells treated with TNFα/CHX (TNF) or left untreated (Ctl). Arrow head indicates L-DNase II. (D) Immunocytochemistry analysis of LEI/L-DNase II (green), PKC zeta (red) and NFkB (green) activation. Nuclei were counterstained with DAPI (blue). Arrow heads indicate intensely stained nuclei. Scale bar represents 10 μm. (E) HeLa cells were transfected with a siRNA against PKC zeta (siRNA) or with a control siRNA (Ctl). The decrease in PKC expression was controlled by Western blot (data not shown). After induction of apoptosis by TNFα/CHX treatment, the rate of cell death was evaluated using the MTT method. The means are significantly different *P* < 0.05, as determined by the test (*). (F) Same experiment as in E but nuclear and cytoplasmic fractions were performed and studied by Western blot for LEI/L-DNase II expression. The presented results correspond to cells transfected with a siRNA against PKC zeta (+) or a control siRNA (−). Arrow head indicates L-DNase II.

The activation of PKC zeta and LEI/L-DNase II in HeLa cells was first evaluated by Western blot (Fig.4A). The treatment with TNFα plus CHX induced PKC zeta cleavage revealed by two new bands (at approximately 50 and 40 kDa) with PKC zeta antibody. This proteolytic degradation has been characterized as a cleavage of native PKC zeta generating two catalytic domains and an increase in PKC zeta activity 29. We did not see an increase in phospho-PKC zeta protein (Fig.4B) after treatment with TNFα/CHX. The activation of LEI/L-DNase II pathway is revealed by an increase in L-DNase II band at 27 kDa in cells treated with TNFα plus CHX compared with control cells (Fig.4C). The activation of this apoptotic pathway has already been described in a model of U937 cells treated with TNFα involving protein AP24 (Apoptotic Protein) 30,31.

To confirm the activation of both proteins, immunocytochemistry was used to verify the subcellular localization of LEI/L-DNase II and PKC zeta (Fig.4D). In normal cells, LEI and PKC zeta had a cytoplasmic localization. In cells treated with TNFα/CHX, we have a nuclear translocation of both proteins. To further confirm the PKC zeta activation, we investigated the status of phospho-NFkB (active form of NFkB pathway) as this transcription factor is one of the substrates of PKC zeta 22,32. We observed an increase in nuclear staining of phospho-NFkB after treatment indicating that the NFkB pathway is also activated.

Taken together these results confirmed that LEI/L-DNase II and PKC zeta pathways were activated in HeLa cells treated with TNFα/CHX.

After validation of the cellular model, we investigated the effect of LEI-PKC zeta interaction on cell survival. We performed PKC zeta specific knockdown assays using siRNA transfection in HeLa cells before treatment with TNFα/CHX. Then we used the MTT assay to estimate the cell viability. As seen in Figure4E, the rate of cell death was increased in PKC zeta siRNA-transfected and treated cells compared with cells transfected with a control siRNA. This result suggests that PKC zeta knockdown had an effect on cell death induced in these conditions of apoptosis.

To verify if this increased cell death is linked to an increase in death induced by the LEI/L-DNase II pathway we performed a nuclear/cytoplasmic fractionation of HeLa cells treated or not with TNFα/CHX for 7 hrs after the PKC zeta knockdown (Fig.4F). In nuclear fractions of TNFα/CHX-treated cells, we confirmed the presence of L-DNase II at 27 kDa, not seen in control (not treated with TNFα/CHX) cells. The PKC zeta knockdown in treated cells induced an increase in nuclear translocation of L-DNase II compared to cells expressing a regular amount of PKC zeta. These results suggested that in this apoptotic condition, the PKC zeta partially impairs the activity of the LEI/L-DNase II pathway by retaining some of the L-DNase II already produced at the cytoplasm.

Taken together all these results suggested that the LEI-PKC zeta interaction had a functional impact on cell survival and that PKC zeta was able to regulate apoptosis induced by LEI/L-DNase II by decreasing its nuclear translocation during apoptosis.

### The effect of PKC inhibition on L-DNase II activity *in vivo*

**Figure 5:**
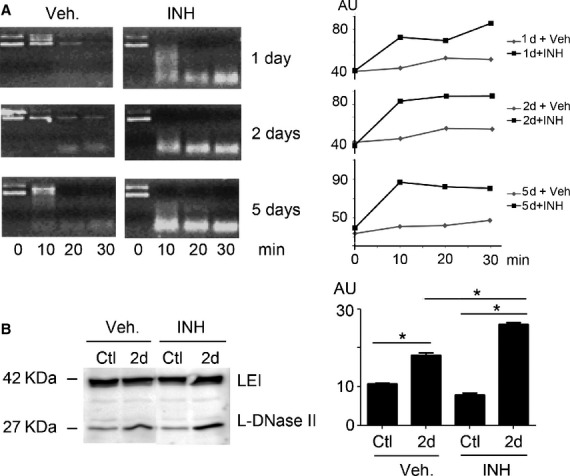
L-DNase II expression and activity in light-exposed rat retinas. (A) Rats were exposed to continuous white light for 2 days (2d) or kept in a normal, cycling light (Ctl). Previous to illumination the inhibitor of PKC zeta was intravitreally injected in the right eye. The left eye was injected with the vehicle. After killing, the retinas were dissected, extracted and analysed for L-DNase II activity using a supercoiled plasmid in ionic DNase II activating conditions. The right panel is a quantification of the lower band (product of degradation). This is a representative experiment out of three. (B) Same experiment as in A but in this case LEI/L-DNase II was analysed by Western blot right panel represent a representative experiment out of four. Right panel represents the quantification of the L-DNase II band; *P* < 0.05 (*).

The results obtained above suggested a protective role of PKC zeta against LEI/L-DNase II-induced apoptosis in a cellular model very different from our original concern. Next step was then to investigate if they were transposable to LIRD. To investigate this issue we injected intravitreally a pharmacological PKC zeta specific inhibitor before light exposure. Then we measured the endonuclease activity of retina total extracts by comparing the injected rats to control rats injected only with the vehicle solution. The endonuclease activity was measured by analysing, on an agarose gel, the degradation rate of a supercoiled plasmid (pHook). Figure5A shows the obtained results. We found an increase in the degradation of the plasmid in the extracts treated with the inhibitor compared to those treated with the vehicle. In retinas from eyes treated with the vehicle solution we saw a progressive but weak increase in L-DNase II activity as time of exposure increased (the lower molecular weight band appeared at 30 min. after 1 day of exposure, 20 after 2 days and 10 for the 5th day). In every case the intravitreal injection on the PKC inhibitor increased this activity.

To confirm this PKC zeta inhibitor effect on LEI/L-DNase II pathway, we investigated the protein pattern of LEI in the retina after injection and light exposure (Fig.5B). At 2 days, Western blot analysis revealed an increase in L-DNase II bands at 42 kDa and at 27 kDa in retina treated with PKC zeta inhibitor compared to retina injected with the vehicle. The intravitreal injection of PKC zeta inhibitor had no impact on the LEI/L-DNase II protein expression in retina non-exposed to light (Ctl) (no difference between non-exposed injected with inhibitor or with vehicle).

Taken together all these results suggested that the PKC zeta inhibition promoted the LEI/L-DNase II pathway by an increase in its endonuclease activity and its nuclear translocation (27 kDa band).

## Discussion

In this study we investigated, for the first time, the putative regulation of LEI/L-DNase II by a kinase involved in a cell survival pathway, the atypical PKC zeta, in a LIRD model. We show that LEI/L-DNase II, effector of cell death in this model, can interact with PKC zeta, and that inhibition of PKC zeta can modulate the pro-apoptotic activity of L-DNase II probably by impairing its nuclear translocation.

We have previously shown that in LIRD photoreceptor cell death is mediated by a caspase-independent pathway 9,10. It seems then interesting to use this knowledge to control and, eventually, slow down degeneration. To get some inside in the regulation of the activity of L-DNase II we investigated its possible interactions in a high output screening. The results obtained drove our attention to PKC zeta, a kinase already identified as possible protection kinase in NMDA-induced stress 21,27. As it was the case for NMDA, PKC zeta was also activated in LIRD. Moreover, as for NMDA stress, this activation could be transiently, although this is not as clear as in the NMDA model.

In NMDA-treated eyes a cleavage of PKC zeta seems responsible for the shutdown of its protective effect 27. Actually, the cleavage of PKC zeta leads to a constitutive activation, to its nuclear translocation and to the induction of apoptosis 27. In LIRD this cleavage was not found, but the increase in its specific inhibitor, PAR-4, may be inactivating the enzyme; leading by a different mechanism to the decrease in its protective effect. The PKC zeta/PAR-4 association is described in many studies where it is shown that the interaction of these two proteins leads to inactivation of the PKC zeta survival pathway through inhibition of the NFkB pathway 33. In our LIRD model, these results suggest that in the early days of illumination, low PAR-4 level allows to maintain the survival pathway in light exposed retinas. However, after 5 days of continuous light exposure, the PAR-4 level increases which allow its interaction with activated PKC zeta resulting in the inhibition of survival pathway and induction of apoptosis. These assumptions were not verified in this model, because it was not our main concern but they are in accord with the existing literature.

Pull-down and co-immunoprecipitation studies indicate that both molecules can interact. *In silico* studies identify a consensus phosphorylation site for LEI/L-DNase II and the mutagenesis of this site, mimicking a phosphorylation, impairs its nuclear translocation. More importantly, when PKC zeta is inhibited by siRNA in a cell model of LEI activation, L-DNase II increases in the nucleus and the survival of these cells is decreased. Although these results were obtained in a cellular model, the increase in L-DNase II expression and activity in light exposed retinas treated with a PKC inhibitor seem to validate, or at least to support the idea that the same process is occurring in the retina. The studies presented here do not permit to say if this interaction is direct or mediated though other molecules, whether these are kinases or not. Further investigation using recombinant molecules *in vitro* should allow to precise this point.

The activation of some kinases during LIRD has already been described. This is the case for the Ceramide kinase such as^34^, phosphoinositide 3 Kinase (PI3 kinase) 35, AKT 36. The data presented in this study show, for the first time, the involvement and activation of PKC zeta in LIRD, adding this protective pathways to other kinases already described as protective in this context. We also show that PKC zeta is able to regulate the LEI/L-DNase II pathway, which is a caspase-independent cell death pathway, so that extending to these type of pathways a property already shown for caspase-dependent apoptosis 21,37. Finally, we show that the LEI/L-DNase II pathway could possibly be regulated by phosphorylation, adding new light in the regulation of this pathway, already shown as regulated by non-caspases proteases 9,30,38,39.

**Figure 6:**
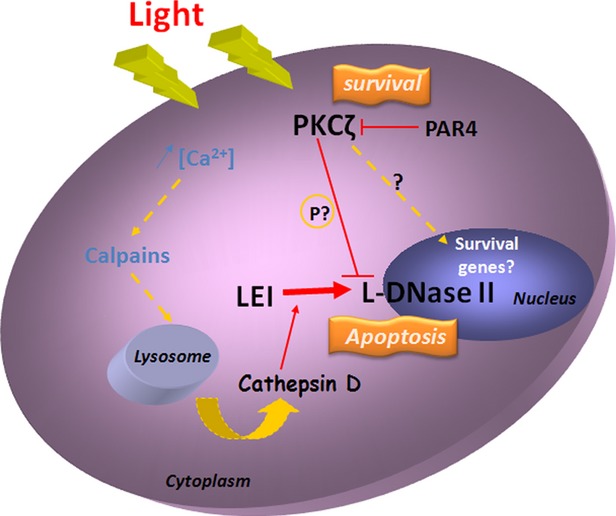
Schematic model of PKC zeta and LEI/L-DNase II in LIRD. Light induces intracellular increase in Ca^2+^ concentration that activates calpains. In turn, calpain 1 permeabilizes the lysosomal membrane. This releases cathepsin D that activates L-DNase II which is translocated to the nucleus to induce photoreceptor cell death. This nuclear translocation is inhibited by PKC zeta to protect the cell. PKC zeta can also activate other anti-apoptotic proteins. Its protective action can be inhibited by its specific inhibitor PAR-4 (*).

This study gives new insight on retinal degeneration, an important issue in the understanding of photoreceptor demise. All together, the data we reported indicate that the events produced in photoreceptors may fit to the model displayed on Figure6. According to this model the intracellular increase in Ca^2+^ concentration induced by light 5 activates calpains that permeabilize the lysosomal membrane by cleaving the lysosomal associated membrane protein 2 39. This permeabilization releases, among other proteases, cathepsin D 9. Cathepsin D activates L-DNase II that translocates to the nucleus to cleave DNA and to induce photoreceptor cell death 9. The nuclear translocation of L-DNase II may be inhibited by PKC zeta by phosphorylation. However, PKC zeta is also an activator of the NFkB pathway, known to have a role in modulating apoptosis through the synthesis of anti-apoptotic factors, an issue that we have not evaluated in this work. Moreover, it has been shown that PKC zeta interacts with p62 (sequestosome) 40 and with the receptor interacting protein kinase 41. These data place PKC zeta at the interface of apoptosis (caspase dependent and independent), oxidative stress response and necrosis, becoming the master of the cell signalling orchestra. In addition, PKC zeta by interacting with PAR-6 (partitioning-defective protein) and the tight junction protein ZO-1 regulates the assembly of tight junctions 42, a structure that can also be modified in LIRD.

In conclusion, we have shown that PKC zeta is activated in LIRD. This activation regulates L-DNase II an apoptosis effector in this degenerative model. Further work is needed to fully characterize the protective effect of this kinase in retinal degenerations.
